# Understanding the impact of congenital infections and perinatal viral exposures on the developing brain using white matter magnetic resonance imaging: a scoping review

**DOI:** 10.1186/s12880-024-01282-9

**Published:** 2024-05-23

**Authors:** Charmaine Natasha Nyakonda, Catherine J Wedderburn, Simone R Williams, Dan J Stein, Kirsten A Donald

**Affiliations:** 1 Department of Paediatrics and Child Health, Red Cross War Memorial Children’s Hospital, University of Cape Town, Cape Town, South Africa; 2 Department of Clinical Research, London School of Hygiene & Tropical Medicine, London, UK; 3 Department of Psychiatry and Mental Health and Neuroscience Institute, University of Cape Town, Cape Town, South Africa; 4 MRC Unit of Risk and Resilience, University of Cape Town, Cape Town, South Africa; 5 Neuroscience Institute, University of Cape Town, Capetown, South Africa

## Abstract

**Background:**

Magnetic Resonance Imaging (MRI)-based imaging techniques are useful for assessing white matter (WM) structural and microstructural integrity in the context of infection and inflammation. The purpose of this scoping review was to assess the range of work on the use of WM neuroimaging approaches to understand the impact of congenital and perinatal viral infections or exposures on the developing brain.

**Methods:**

This scoping review was conducted according to the Arksey and O’ Malley framework. A literature search was performed in Web of Science, Scopus and PubMed for primary research articles published from database conception up to January 2022. Studies evaluating the use of MRI-based WM imaging techniques in congenital and perinatal viral infections or exposures were included. Results were grouped by age and infection.

**Results:**

A total of 826 articles were identified for screening and 28 final articles were included. Congenital and perinatal infections represented in the included studies were cytomegalovirus (CMV) infection (*n* = 12), human immunodeficiency virus (HIV) infection (*n* = 11) or exposure (*n* = 2) or combined (*n* = 2), and herpes simplex virus (HSV) infection (*n* = 1). The represented MRI-based WM imaging methods included structural MRI and diffusion-weighted and diffusion tensor MRI (DWI/ DTI). Regions with the most frequently reported diffusion metric group differences included the cerebellar region, corticospinal tract and association fibre WM tracts in both children with HIV infection and children who are HIV-exposed uninfected. In qualitative imaging studies, WM hyperintensities were the most frequently reported brain abnormality in children with CMV infection and children with HSV infection.

**Conclusion:**

There was evidence that WM imaging techniques can play a role as diagnostic and evaluation tools assessing the impact of congenital infections and perinatal viral exposures on the developing brain. The high sensitivity for identifying WM hyperintensities suggests structural brain MRI is a useful neurodiagnostic modality in assessing children with congenital CMV infection, while the DTI changes associated with HIV suggest metrics such as fractional anisotropy have the potential to be specific markers of subtle impairment or WM damage in neuroHIV.

**Supplementary Information:**

The online version contains supplementary material available at 10.1186/s12880-024-01282-9.

## Introduction

The maternal *in utero* environment provides foundational support for the fetus’ physical and brain development. Congenital and perinatal viral infections may therefore have important long-term effects on a child’s development and health. This group of infections are known to contribute to long-term neurodevelopmental disability among children worldwide, particularly in low – middle income countries (LMICs) where the burden of infectious disease is high and access to medical services is limited [1]. Cytomegalovirus (CMV), human immunodeficiency virus (HIV), rubella, herpes simplex virus (HSV), varicella zoster virus, enteroviruses and Zika virus are examples of viral agents described as having the potential to cause congenital and perinatal infections in infants which may affect the central nervous system (CNS) [2–4].

Populations in LMICs face a higher overall burden of infectious diseases and exposures. In particular, Africa accounts for a large proportion of the global burden of HIV infection [5, 6]. CMV is a leading cause of congenital viral infection and long-term neurodevelopmental disabilities among children [7, 8]. Despite the high disease burden in LMICs, CMV infection is frequently under-reported due to limited screening and tools for assessment in the early years of life [8]. Given the higher burden of congenital and prenatal infections in LMICs, it is crucial to understand how these infections may impact brain development. Assessing whether neuroimaging can support diagnostic approaches, and if the observed injury persists over time may aid in understanding pathways through which these infections impact long-term neurological and developmental outcomes. Further, in the clinical setting, accurate diagnosis and prognosis is key to inform treatment and prevent or mitigate morbidity.

Studies have demonstrated that Magnetic Resonance Imaging (MRI) has become a valuable tool for investigating paediatric brain development and has enabled researchers to gain a better understanding of the structure, function, and connectivity of the developing brain [9]. Diffusion-weighted and diffusion tensor MRI (DWI/ DTI) is an advanced MRI imaging modality which has enabled researchers to assess white matter (WM) microstructural integrity in vivo and visualize WM fibres [10]. Unlike structural MRI which describes the volume and location of WM, DTI provides a more detailed assessment of WM microstructure through assessing the measurement and directionality of water molecules [11, 12]. Therefore, DTI/DWI may be useful in detecting more subtle cerebral injury which affects WM tracts. DTI outcome measures also known as DTI metrics include fractional anisotropy (FA), mean diffusivity (MD), radial diffusivity (RD) and axial diffusivity (AD). FA measures water molecule diffusion anisotropy and is used as a measure of overall integrity of white matter. MD is the average diffusion magnitude of water molecules in a given brain region, where changes in MD are thought to be indicative of alterations in tissue microstructure. RD reflects the diffusion of water molecules perpendicular to the primary axis axonal while AD measures diffusivity along the primary axis of diffusion, providing measures of the integrity of white matter myelin and axonal membranes respectively [11, 13]. In adult literature, healthy white matter tends to demonstrate higher FA and low MD, although this may differ in paediatric populations where white matter is developing.

The DTI/DWI magnetic resonance diffusion approaches have allowed researchers to further explore the sensitivity of specific WM tracts to inflammation and infection. For example, WM has shown high sensitivity to HIV infection with alteration in the DTI metrics particularly in key central WM tracts [14]. Emerging research suggests that DTI is the most sensitive and reliable imaging technique in detecting subtle alterations to WM and detecting infection-induced WM microstructural alterations [15], even in patients whose tissue appeared normal on structural MRI scans [16]. However, most literature has been on adults and there is a need to understand the impact of congenital viral infections and exposure on WM in the developing brain. In vivo DTI/DWI can potentially help in the diagnosis, management and understanding of pathophysiology. Advances in imaging in recent years allow a more extensive focus on white matter in paediatric populations. The primary aim of this scoping review was to assess the range of work on WM neuroimaging approaches to understanding the impact of congenital and perinatal viral infections or exposures on the developing brain.

## Methodology

Literature comparing systematic and scoping reviews often advise the use of a systematic review when the aim of the review is to critically appraise available literature by addressing questions related to feasibility, acceptability, and effectiveness [17]. While scoping reviews are more advisable when reviewers are seeking to scope the literature and provide a general overview of the currently available literature and evidence [17].Therefore, given that the main aim of this review was to scope and examine current literature, and also identify any gaps in knowledge, a scoping review was conducted rather than a systematic review. This review was conducted according to the framework laid out in the Arksey and O’ Malley methodological guide for scoping reviews, including five stages: identifying the research question, identifying relevant studies, study selection, data charting, results collating, summarizing, and reporting [18]. Our approach also included the optional sixth stage of the Arksey and O’Malley framework, which involved consulting with research experts who offered relevant insights on research strategy development, database sources and clinical relevance.

### Data sources and search strategy

The search strategy included three electronic databases: Web of Science (1991 – January 2022), Scopus (1986 – January 2022) and PubMed (1979 – January 2022). The search query included: WM imaging, WM magnetic resonance imaging, WM MR imaging, congenital and perinatal infection, or exposure (See Additional file 1). Reference lists of relevant articles were manually searched to identify any additional relevant articles. There were no limits placed on publication year, subject areas, source titles, geographic location, study design and categories on the database searches.

### Inclusion and exclusion criteria

Articles were eligible for inclusion if they were peer-reviewed and primary research papers, assessed congenital and perinatal viral infections or exposures, and if they described applications of WM imaging techniques. The eligible participant population included children, aged between birth − 18 years old. Animal studies and articles written in languages other than English were excluded. Studies which imaged the effects of medical conditions other than congenital and perinatal viral infections or exposures were excluded. Studies which imaged the effects of other comorbidities, secondary to congenital and perinatal viral infection or exposures were also excluded. A second reviewer (SW) independently reviewed the included articles to verify that they fully met the inclusion criteria.

### Data summarizing and reporting

In this scoping review we report on studies representing a number of viral infections and exposures, and reporting on qualitative and quantitative findings in the exploration of the impact of these infectious exposures on the developing brain using WM imaging. Our format on reporting the DTI or structural MRI findings, divided the 28 articles into 3 age ranges consisting of findings at birth – 3 years, 4–8 years and 9–18 years (Tables 1 and 2). In the early years of development from birth, the greatest brain growth occurs, and this changes during this period may have critical implications for long term brain health and development. Given the trajectory of white matter growth and maturation over these years, we present findings grouped by age range. The extensive scoping review research strategy including the data collating, summarizing and reporting strategy is described in Additional file 1.

**Table 1:** Demographics overview of included studies

Reference	Country	Congenital infection / Perinatal viral infection or exposure	Neuroimaging time-point	Participants, (*n*) = sample size	WM Imaging modality
[19]	Italy	CMVI	1 month, with a scheduled 6 year follow up	CMVI; *n* = 40	Structural MRI
[20]	Japan	CMVI	within 3 months from birth	CMVI(+ clinical symptoms): *n* = 23 CMVI (- clinical symptoms): *n* = 19	Structural MRI
[21]	Italy	CMVI	5–54 months	CMVI: *n* = 14	Structural MRI
[22]	Belgium	CMVI	1–78 days	CMVI(+ clinical symptoms): *n* = 26 CMVI (- clinical symptoms): *n* = 165	Structural MRI
[23]	UK	CMVI	birth – 4 months	CMVI(+ clinical symptoms): *n* = 36 CMVI (- clinical symptoms): *n* = 35	Structural MRI
[24]	Netherlands	CMVI	40.4–41.7weeks	CMVI: *n* = 21 CMV uninfected: *n* = 61	Structural MRI, DTI
[25]	Japan	CMVI	63–127 months	CMVI(bilateral): *n* = 5 CMVI(unilateral): *n* = 4 *Unilateral and bilateral hearing disturbances*	Structural MRI
[26]	USA	CMVI	22–299 days	CMVI : *n* = 17 *Population with a range of hearing loss	Structural MRI
[27]	Korea	CMVI	2- 375 days	CMVI(+ clinical symptoms): *n* = 31	Structural MRI
[28]	Belgium	CMVI	Birth – 6 years	CMVI : *n* = 639	Structural MRI
[29]	Japan	CMVI	4months- 13 years	CMVI : *n* = 31	Structural MRI
[30]	Italy	CMVI	2days – 3 years	CMVI(+ clinical symptoms): *n* = 27 CMVI (- clinical symptoms): *n* = 17	Structural MRI
[31]	South Africa	HIV and HEU	7 years	HIV+: *n* = 65 HEU: *n* = 19 HUU *n* = 27	DTI
[32]	South Africa	HIV	9–11 years	HIV+: *n* = 204 Matched controls: *n* = 44	Structural MRI, DTI
[33]	South Africa	HIV	5 years	HIV+:*n* = 38 Controls: *n* = 11	DTI
[34]	South Africa	HIV	9–12 years	HIV+ : *n* = 168 Controls : *n* = 43	DTI
[35]	India	HIV and HEU	8–15 years	HIV+ : *n* = 22 HEU: *n* = 18 HUU: *n* = 8	DTI
[36]	South Africa	HIV on ART	9–11 years	HIV+(ART < 2years): *n* = 46 HIV+ (ART > 2years): *n* = 79	DTI
[37]	Netherlands	HIV on cART	8–18 years	HIV+ : *n* = 28 Controls : *n* = 34	Structural MRI, DTI
[38]	Thailand	HEU	5–15 years	HEU: *n* = 30 HUU : *n* = 33	Structural MRI, DTI
[39]	South Africa	HIV	5 years	HIV+: *n* = 39 Controls : *n* = 13	DTI
[40]	South Africa	HIV	6–16 years	HIV+: *n* = 75 Controls : *n* = 30	DTI
[41]	China	HIV	12–18 years	HIV+ : *n* = 15 Controls : *n* = 26	DTI
[42]	South Africa	HEU	2–4 week old	HEU : *n* = 15 Controls : *n* = 24	DTI
[43]	South Africa	HIV	6–15 years	HIV+ : *n* = 50	DTI
[44]	South Africa	HIV	8–12 years	HIV+: *n* = 12 Healthy controls: *n* = 12	DTI
[45]	South Africa	HIV	8–54 months	HIV+: *n* = 44	Structural MRI
[46]	Japan	HSV	17 days – 76 months	HSV + patients: *n* = 32	Structural MRI, DWI, DTI

**Key**: ART: Antiretroviral therapy, cART: Combined antiretroviral therapy, CMVI: Cytomegalovirus infection, HIV: Human Immunodeficiency Virus, HEU: HIV exposed and uninfected, HSV: herpes simplex virus, + : infected, DTI: Diffusion tensor Magnetic Resonance Imaging, DWI: Diffusion-weighted Magnetic Resonance Imaging, MRI: Magnetic resonance imaging , WM: White matter

## Results

**Fig. 1:**
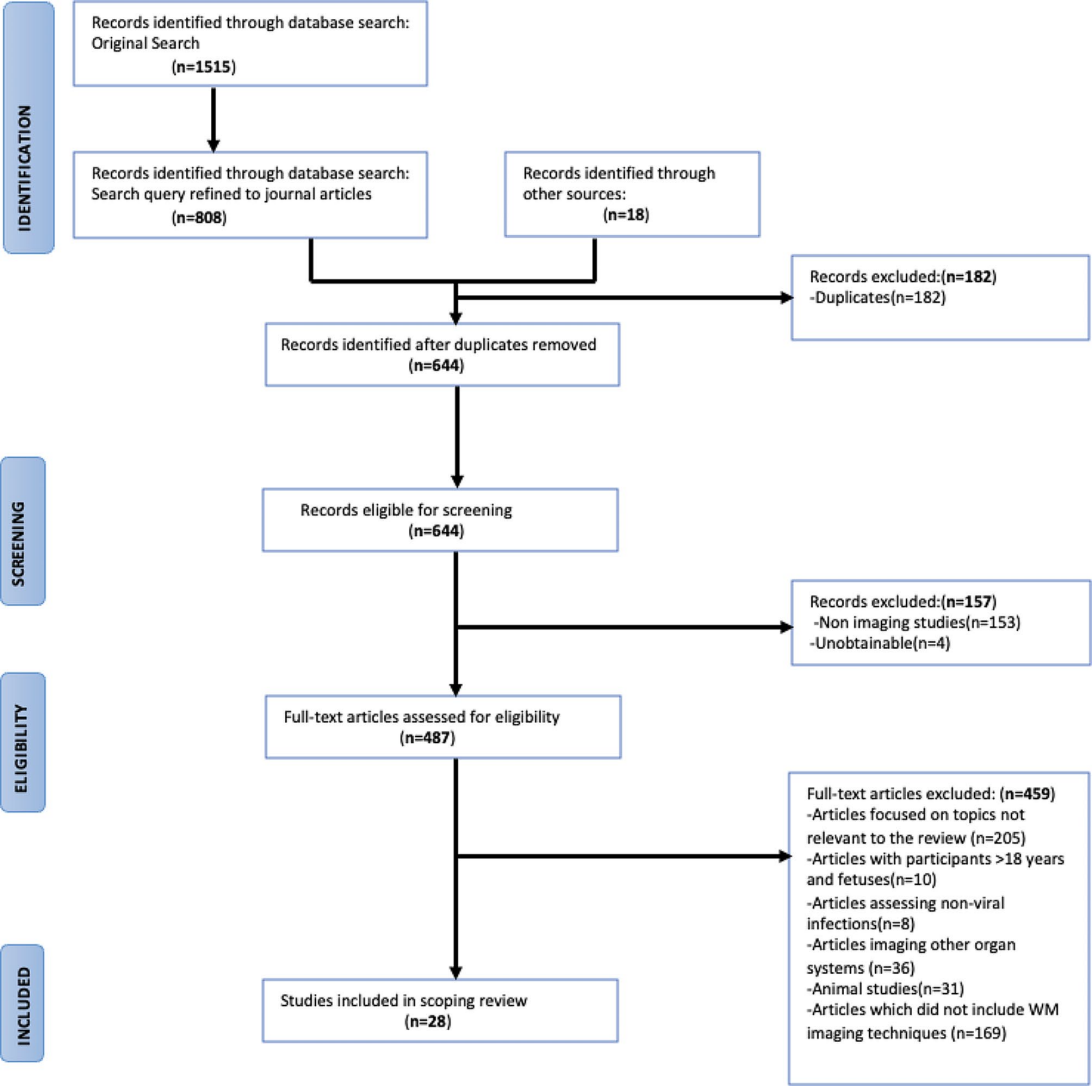
Identification of scoping review studies via databases and other sources

The initial database and additional snow-balling search strategy yielded 1515 articles, including all article types (books and documents, clinical trials, meta-analysis, case studies and journal articles). Of these, 826 journal articles were identified for screening after refining the search query to journal articles only (Fig. 1). The final 826 articles were screened for duplicates, type of imaging study, journal accessibility. After an assessment for eligibility, 459 articles were excluded primarily because the articles focused on topics which were not relevant to the review or did not include WM imaging modalities, and a total of 28 articles (Tables 1 and 2) were included in this review (Fig. 1; Table 2).

A total of 12 articles reported on neuroimaging in children congenital CMV, 11 articles on those with perinatal HIV, two articles reported on groups of children with either perinatal HIV or who were HIV exposed uninfected (HEU), two articles on HEU specifically, and one article reported on neuroimaging in the context of congenital HSV.

Studies investigating the neurological outcomes of children living with HIV infection or HIV exposure without infection, were largely conducted in LMICs, whereas most congenital CMV studies were conducted in upper-middle income to high-income countries as indicated in (Table 1). The included articles overall had a median study sample size of 49 participants and an average sample size of 90 participants, ranging from *n* = 9 to *n* = 639.The included articles had a median neuroimaging time point of four years of age, where CMV articles had a median of seven years, and the remaining articles consisting of HIV and HEU studies had a median of 10 years.

**Table 2:** Affected regions, DTI/DWI and structural MRI findings

Median Neuroimaging time point (months)		Reference		Country	Congenital infection / Perinatal infection or exposure	Affected Regions	DTI / structural MRI finding
	Findings at birth – 3 years						
1			[19]	Italy	CMVI	Temporal lobe	WM hyperintensities, polymicrogyria and cysts
3			[20]	Japan	CMVI	Periventricular regions	WM hyperintensities, ventriculomegaly and cysts
29,5			[21]	Italy	CMVI	Temporal lobe	WM hyperintensities, periventricular cysts, and cortical abnormalities
						Parietal lobe	WM hyperintensities, periventricular cysts
1,3			[22]	Belgium	CMVI	Cerebellar	WM hyperintensities, cysts
						Temporal	Subependymal cysts
						Frontal WM, Anterior temporal region	WM hyperintensities
2			[23]	UK	CMVI	Biparietal, parietotemporal	WM hyperintensities
						Temporal, frontoparietal, frontotemporal	WM hyperintensities, cysts
9,4			[24]	Netherlands	CMVI	Frontal parietal and occipital regions	↓ FA ; ↑ MD, AD & RD
5,3			[26]	USA	CMVI	Temporal lobe	WM hyperintensities, polymicrogyria, parenchymal cysts
6,2			[27]	Korea	CMVI	Cerebellar	WM hyperintensities, subependymal cysts, polymicrogyria, calcification
36			[28]	Belgium	CMVI	Cortical region	Gyration disorders, polymicrogyria, cortical atrophy, subependymal cysts and WM hyperintensities
18			[30]	Italy	CMVI	Periventricular region	WM hyperintensities, cysts and myelination delay
11,5			[35]	India	HIV	Cingulum	↓FA
						Cerebellar	↓FA
						Left fusiform	↓FA
						Right corpus callosum	FA -r with verbal learning
						Left insula	FA -r with verbal learning
						Left hypothalamus	FA -r with verbal learning
					HEU	Cerebellum crus1	**-**↓MD
						Gyrus	↓ FA
						Parietal lobe	↑FA
0,5			[42]	South Africa	HEU	Middle cerebellar peduncles	↑FA
						Left hippocampal cingulum	MD -r with Dubowitz abnormal neurological signs score
						Left UF	FA + r with Dubowitz abnormal neurological signs score
31			[45]	South Africa	HIV	Deep WM, frontal regions, parietal regions, subcortical WM	WM hyperintensities
38			[46]	Japan	HSV	Cerebral region, cortical region	WM Hyperintensities
	Findings at 4–8 years						
95			[25]	Japan	CMVI	Frontal lobe, parietal lobe occipital lobe and temporal lobe	WM hyperintensities
80,4			[29]	Japan	CMVI	Deep WM, parietal lobes, anterior temporal	WM hyperintensities,
						Fronto-parietal lobe	WM hyperintensities, polymicrogyria
						Fronto-parieto-temporal lobe	WM hyperintensities, polymicrogyria
84			[31]	South Africa	HIV	ILF	↓FA
						IFOF	↓FA, ↑MD
					HEU	CST	↓MD
						Right posterior corona radiata	↑FA
60			[33]	South Africa	HIV	Left forceps minor	↑AD $\sim$ ↑ practical reasoning scores
						Right UF	RD -r with performance subscale scores
						CST	AD -r with motor coordination
60			[39]	South Africa	HIV	CST	↓FA
	Findings at 9–18 years						
120			[32]	South Africa	HIV	Left superior cerebellar peduncle, internal capsule, right superior corona radiata, left sagittal stratum, left SLF, right SFOF	↓FA
126			[34]	South Africa	HIV	whole brain WM volumes	Lower brain volumes and cortical thickness
120			[36]	South Africa	HIV	Superior corona radiata	↓FA, ↑MD
156			[37]	Netherlands	HIV	Basal ganglia	↑CBF in WM
120			[38]	Thailand	HEU	Cingulum, Internal capsule, optic/temporal region, uncinate, thalamic radiation	RD & MD **-**$\sim$ Full & Performance IQ
132			[43]	South Africa	HIV	Left cerebellar peduncle, corpus callosum, fornix, internal capsule	↓FA
180			[41]	China	HIV	Corpus callosum, corona radiata, frontal & parietal WM, left SLF	↓FA
126			[40]	South Africa	HIV	Fornix, cerebellar peduncles, FOF, cingulum	↓FA
						Fornix, cerebellar peduncles, FOF, cingulum, SLF	↑MD
120			[44]	South Africa	HIV	Corpus callosum	↓FA, ↑RD
						SLF	↑MD

Key: AD: Axial Diffusivity, ART: Antiretroviral therapy, cART: Combined antiretroviral therapy, CMVI: Cytomegalovirus infection, CST: Corticospinal Tract, DTI: Diffusion tensor Magnetic Resonance Imaging, DWI: Diffusion-weighted Magnetic Resonance Imaging, FA: Fractional Anisotropy, HIV: Human Immunodeficiency Virus, HEU: HIV exposed and uninfected, HSV: herpes simplex virus, IFOF: Inferior Fronto-Occipital Fasciculus, ILF: Inferior Longitudinal Fasciculus, MD: Mean Diffusivity, MRI: Magnetic resonance imaging, RD: Radial Diffusivity, UF: Uncinate Fasciculus, WM: White matter, - : negative, ↓: lower, ↑: higher, -$\sim$ : negative association. $\sim$: association. r : correlation, -↓: decreased

### The impact of congenital and perinatal infections and exposures on the developing brain in paediatric populations at different ages

In the early years of paediatric brain development, the greatest brain growth occurs and this period has critical developmental implications. Given the developmental trajectories of white matter, we present findings grouped by age range.

#### Median age of birth through 3 years

Among the studies assessing children aged between birth − 3 years, congenital CMV infection was the most represented with 10/14 studies (71%). In CMV infection and HSV infection WM hyperintensities were the most frequently reported MRI finding. In CMV infection the WM hyperintensities were either confluent, bilateral or multifocal (Table 2). The most commonly reported location for WM hyperintensities in congenital CMV infections (> 50%), was the temporal region, though similar changes where documented in frontal, parietal, and occipital regions less consistently across studies.

The studies investigating children with HIV infection reported a range of regions with group differences in diffusion metrics including frontal, parietal, cerebellar, uncinate fasciculus (UF), fusiform, corpus callosum and other regions noted in Table 2. In children who were HEU, the cerebellar region was the most commonly reported region with diffusion metric group differences.

#### Median age of 4–8 years

This age window had the least number of neuroimaging studies, likely due to the difficulty in neuroimaging in this age group (Table 2). Among the six studies in this age window, children with perinatal HIV infection [31, 33, 39] and CMV infection [25, 29] were equally represented, while a single study reported neuroimaging findings in children with HIV alongside children who were HEU [31].

The affected regions reported in children with congenital CMV infection in this age-group, included the parietal, temporal and frontal lobes (Table 2). Structural MRI findings in this group of children included WM hyperintensities as the most commonly reported abnormality, similar to the younger age group.

Findings reported in children with perinatal HIV infection included diffusion metric group differences in regions including corticospinal tract (CST), UF, forceps minor, inferior longitudinal fasciculus (ILF), Inferior Fronto-Occipital Fasciculus (IFOF) and corona radiata (Table 2).

**Fig. 2:**
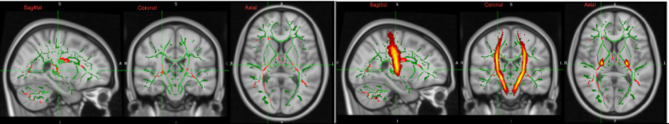
Visualisation of the corticospinal tract highlighted on a DTI skeleton. Images showing visualization of the CST region FA, along 3 different planes. L-R denotes radiological orientation where L = left, R = right, A = anterior, P = posterior, S = superior and I = inferior. The*se sample images were* generated using the JHU white-matter tractography atlas in the FMRIB Software Library (FSL) software and is shown as a red-orange overlay. * Images are for visualization of the CST purposes only, images do not depict any clinical outcomes*

Findings reported in children who are HEU included diffusion metric group differences in regions including the CST and corona radiata (Table 2). The CST (Fig. 2) was also the most commonly reported region with diffusion metric group differences in children with HIV infection at this age in children as well as in those who were HEU.

#### Median age of 9–18 years

**Fig. 3:**
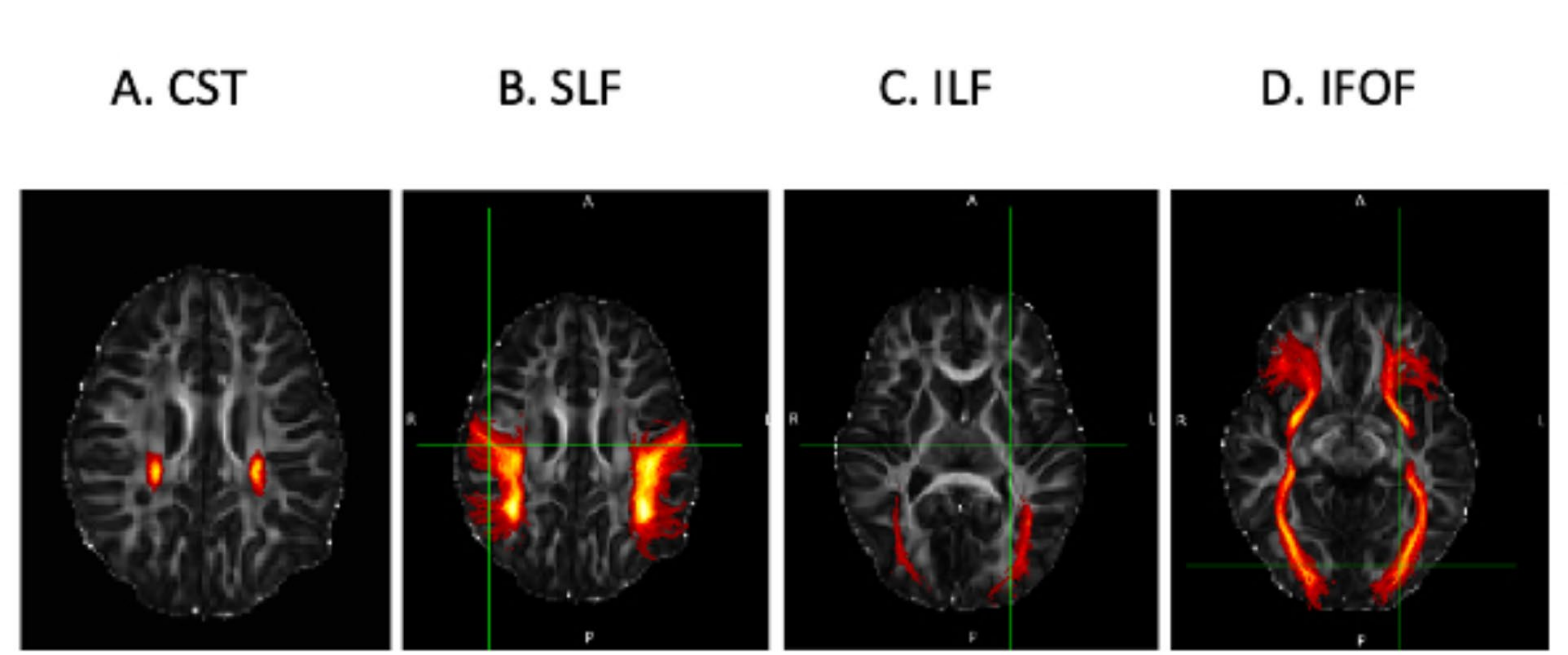
Visualization of Association and Projection WM tracts highlighted using the JHU WM Tractography Atlas. Images depict 3D axial plane images of the CST, SLF, ILF and IFOF white matter tracts. The tract images were created using the FMRIB Software Library (FSL) software. Sample images of Each of the 4 tract regions were created using the JHU white-matter tractography atlas within FSL and are shown as a red-orange overlays on orthogonal slices through the brain. *Images are for visualization of the CST, SLF, ILF and IFOF purposes only, images do not depict any clinical outcomes*

**Fig. 4:**
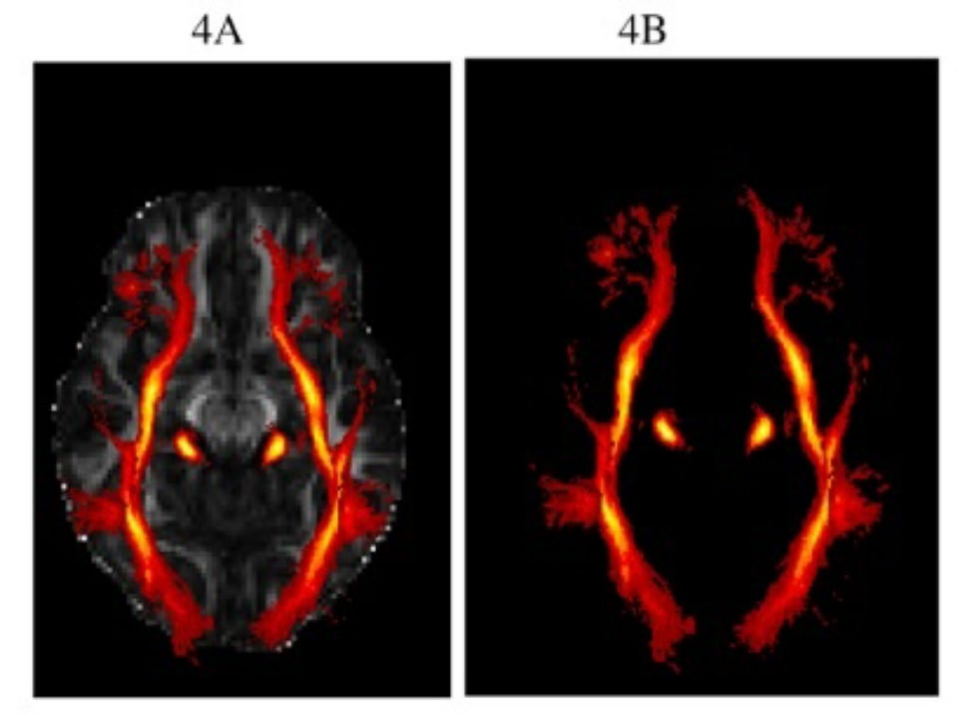
Visualization of the combined projection and association WM tract highlighted using the JHU WM tractography atlas. Images depict 3D axial plane images of the association and projection fibre white matter tracts SLF, ILF, IFOF, SFOF and CST in a single diagrammatic representation. Figure 4a, b were both created using FMRIB Software Library (FSL) software. Sample images of each of the white matter tract regions were created using the JHU white-matter tractography atlas within FSL and are shown as a red-orange overlays on orthogonal slices through the brain in Fig. 4a, b shown as simple red-orange overlays on the FSL working space.*Images are for visualization of the projected and association WM tracts purposes only, images do not depict any clinical outcomes*

In the age range 9–18 years there were no articles on CMV so only articles assessing HIV and HEU are described. Similarly to imaging outcomes in 4–8-year-olds, in this older age window, the cerebellar region was the most commonly reported region to have diffusion metric group differences in both children who were HIV infected and those who were HEU, compared to controls. Overall in this older age window, more than 50% of the studies reported diffusion metric differences of the corpus callosum in both children with perinatal HIV infection and HEU when compared to controls. More than half of the HIV studies reported diffusion metric group differences in one or more of the Association tracts (Figs. 3 and 4) in children with perinatal HIV infection compared to healthy controls. Although there were no reported group differences in HEU compared to controls in these tracts, the HEU study reported negative associations between diffusion metrics including RD and MD in Association fibre WM tracts and full performance Intelligence Quotient(IQ).

In all 3 age ranges, structural MRI sequences were more common in studies assessing CMVI, while studies assessing the impact of HIV and HEU more commonly used DTI, or a combination of structural MRI and DTI. The implications of findings per imaging modality are further discussed below.

## Discussion

In this scoping review we report on studies representing a mixture of qualitative and quantitative approaches in the exploration of the impact of congenital infections and perinatal viral infections or exposures on the developing brain using WM imaging. The studies using qualitative methods focused on use of neuroimaging changes as diagnostic or prognostic markers and the quantitative studies looked at patterns of injury between groups of children who were exposed or infected with these viruses, potentially providing new insights into general underlying mechanisms.

### Diagnostic value of WM Imaging techniques

Studies focusing on using structural MRI in a qualitative manner to assess the neurological impact of congenital infections and perinatal viral infections on WM described a range of pathological findings. Out of the 28 included studies (Table 1), 16 used structural MRI with qualitative analysis approaches. CMV was the only infection for which there were studies that specifically reported on the potential diagnostic or prognostic value of WM imaging in congenital and perinatal viral infections. Overall, studies investigating children with congenital CMV demonstrated heterogenous type and location of WM hyperintensities. These findings were most evident in the critical age period between birth – 2 years when it is typically more difficult to detect brain imaging abnormalities and when opportunities are best for early intervention [20].

**Fig. 5:**
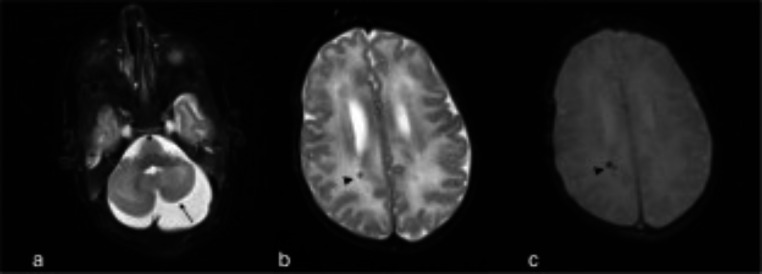
Clinical example of the use of brain MRI in CMV infection: Adapted from Vande Walle and colleagues [22]. Clinical example of the use of brain MRI in CMV infection: “Brain MRI in a 3-week-old male with confirmed cCMV infection, hematologic abnormalities, clinical signs of CMV infection, and hearing loss. Axial T2-weighted image (**a**) showing asymmetric, hypoplastic cerebellar hemispheres (arrow). Axial T2- (**b**) and T2*-weighted (**c**) images showing hypointense punctate lesions (arrowhead) near the posterior wall of the right lateral ventricle, with blooming artifact on the T2* image, representing calcifications.” Figure taken from Fig. 2 [22] in Vande Walle C et al., 2021, Eur Radiol. Oct;31 [10]:8001-801 [22]. Copyright 2021. European Society of Radiology. Reprinted with permission from Springer Nature (License : 5,752,021,193,227)

In their paper, Vande Walle and colleagues [22] included a clinical case study of a brain MRI for a 3-week-old male with confirmed cCMV infection. The patient presented with hematologic abnormalities, clinical signs of CMV infection, and hearing loss [22] (Fig. 5). In their paper and analysis, they suggested that the use of MRI may have value in determining management and prognosis and for counselling parents of children with CMV infection [22]. Of particular note is that in their analysis they examined abnormalities using MRI in both both symptomatic and asymptomatic congenital CMV infection [22]. Their findings in relation to this clinical case showed that symptomatic children with congenital CMV infection were most likely to show abnormalities on brain MRI and WM lesions were the most commonly detected lesions [22].

The high sensitivity for identifying WM hyperintensities makes structural brain MRI a useful neurodiagnostic modality in assessing children with both symptomatic and asymptomatic congenital CMV infection. Study findings also suggest that structural MRI is better than cranial crUS and CT scans at showing mild abnormalities [19, 20, 27]. A study of children with congenital CMV from Italy suggested that structural MRI findings can better predict risk of long-term sequelae than the presence of symptoms at birth [30]. This group also suggested that MRI scores were a valuable diagnostic tool in the early prediction of neurological impairment in assessing asymptomatic and symptomatic children with CMV [30]. A study comparing applications of structural MRI and cranial ultrasound imaging in children with CMV suggested that structural MRI is useful in characterizing brain abnormalities and may serve as a useful complementary tool to cranial ultrasound in paediatric populations aged between birth − 6 years [28].

A single study demonstrated that the use of lesion progression assessment in DTI/DWI WM imaging was useful in early diagnosis of neonatal HSV encephalitis [46]. It was the only study in the scoping review where congenitally infected infants were imaged and examined at different time points (Tables 1 and 2). The study findings suggested that DTI/DWI were the most sensitive techniques to reveal cortical hyperintensities in early periods of neonatal HSV encephalitis onset and could be used as indicators for treatment initiation or continuation given the absence or presence of cortical hyperintensities, and the outcomes of progressive lesion assessments at different time points [46]. However, more studies assessing congenitally infected and perinatally infected or exposed children at different time points are needed to validate the findings suggesting that lesion progression assessment through DTI/DWI is useful in early diagnosis and determining treatment plans in children with HSV and other congenital viral infections or exposures. Considering that most of the studies focusing on congenital CMV infection were based in upper-middle income to high-income countries, findings in this scoping review suggest that there is still a gap in the literature focused on examining the neurodevelopmental trajectory of children with congenital CMV infection in LMICs. Given the high burden of CMV infection in LMICs, more cohort studies assessing CNS involvement in congenital CMV infection are needed in LMICs contexts. The limited access to MRI imaging in LMICs can be attributed to the high acquisition and maintenance cost of conventional high-field MRIs, lack of appropriate infrastructure to meet high-field MRI safety standards and the limited availability of radiographers with expertise to use the high-field MRI unit [47]. The emerging innovative low-field MRI could be useful in expanding CMVI and HEU focused neuroimaging research in LMICs because it is low cost and more affordable than the high field MRI. Emerging research studies have managed to use low field MRI to successfully acquire structural MRI data [48, 49]. However, despite successful structural MRI data acquisition on ultra low field MRIs, high field MRIs have an impressive range of imaging sequences and acquisition protocols which are not available or matched in low field MRIs to date [48, 49]. Although research on ultra low field MRIs shows great promise, further validation studies comparing the accuracy of ultra low field MRI data to high field MRI data are still needed.

#### White matter damage to specific regions identified in the three different age groups on DTI

A number of brain regions have been implicated in the impact of specific viral infections and exposures on the developing brain at different ages. Most of the included studies assessed the associations between regions with diffusion metric group differences and neurocognitive and behavioural assessments, reporting that both children with HIV infection and children who were HEU exhibited motor, language and visual deficits (Table 2) [33–37, 42, 44]. Some of the regions of associated with neurocognitive and behavioural assessment outcomes included the CST, corpus callosum, hypothalamus and left insula in children with HIV infection (Table 2) [33, 35] and the cerebellar region, UF and cingulum in children who were HEU.

The corticospinal tract is known to be important for voluntary motor function [50].The corpus callosum plays an important crucial role in signal transmission across the right and left hemispheres, and previous DTI studies have suggested that the corpus callosum has a non-linear developmental trajectory [51].While the cerebellar region is important for cognitive, motor and behavioural tasks, and damage to this region has been links to deficits in motor and cognitive skills [52, 53]. In HIV infection cerebellar damage has been linked to deficits in verbal learning [35]. In HEU children, cerebellar damage has been linked to deficits in neurological outcomes assessed using the Dubowitz neurological examination in neonates [42]. Although the effects of HIV infection on white matter are well-documented, there are a range of factors affecting brain development in children with HIV and children who are HEU which include the influence of maternal health, immune regulation and in utero environment inflammation, and evidence suggests that some of the underlying mechanisms of injury may overlap.

Association fibre damage or deterioration has previously been attributed to a deterioration of axonal architecture, loss of myelin membrane integrity and loss of organization in WM tracts [45, 54]. The IFOF, Superior Fronto-Occipital Fasciculus (SFOF), Superior Longitudinal Fasciculus (SLF), UF and ILF (Figs. 3 and 4) are long association fibres which interconnect cortical regions within the same cerebral region. The UF connects limbic regions in the temporal lobe to the frontal lobe [55] and damage to this tract, has been linked to deficits in motor coordination in HIV infection and HEU [33, 56, 57]. The IFOF connects the parietal and occipital lobes to the frontal lobe [58], and damage to this tract has been linked to language deficits and previous studies suggest that it is an essential contributor to the language network and plays a role in the visual recognition system [35, 59]. The SFOF connects the frontal, parietal and occipital lobes and plays a role in spatial awareness and visual processing and the SLF connects the parietal, occipital and temporal lobes with ipsilateral frontal cortices and damage to this tract has also been linked to language deficits [60–62]. The ILF connects the occipital and temporal-occipital regions of the brain to the anterior temporal regions [56, 63]. Neurological insult related damage to the ILF tract has been linked to visual cognition deficits in children living with HIV [33].

The WM microstructural integrity of the association fibres is clinically relevant in congenital infections and viral perinatal infections or exposure as evidenced by the association of altered WM in these tracts with poorer information processing, executive function, memory resulting in motor and language deficits reported [33–35, 44, 46]. Overall, findings from this review suggest that damage to the identified tracts (Table 1) may contribute to neurodevelopmental impairment and that the number and the size of WM hyperintensities was associated with the type of neurodevelopmental impairment present [19–22, 26, 27, 30, 33, 34].

#### Important considerations when assessing scoping review findings at the 3 different age-groups

The articles included in the scoping review covered a broad age range (birth – 18 years), however, articles covering the age range of birth – 3 years and 9–18 years were the most frequently represented (Table 2). These scoping review findings suggest that there is limited literature available on the effect of congenital and perinatal infections or exposures on WM integrity of paediatric populations during the age window of 4–8 years old. Although most rapid WM development occurs in the first 3 years of life, previous studies have demonstrated that WM development during the neonatal period is consistently predictive of WM tract average values in later years including the 4–8year age range [64]. In addition, emerging study findings have suggested that after the age of 2 years, brain development is characterized by processes including plasticity, reorganization and remodelling of major networks and circuits which were already established during rapid WM microstructural development [65]. Therefore, WM imaging studies in the 4–8-year range represent an important stage of WM microstructure development and provide additional context and information on the process of WM development during a time period when WM microstructural major networks and circuits are maturing.

To date, congenital infection research has supported the hypothesis that the gestational age at the time of insult (infection or exposure) is an important factor in determining the pattern of CNS injury [66–68]. Gestational age determines the pattern of CNS injury because neuronal formation occurs between 8 and 24 weeks with neuronal migration until 24–26 weeks, astrocyte generation begins near the end of neuronal production and oligodendrocytes are produced during the third trimester into early postnatal life [68]. Oligodendrocytes are important for myelination, while astrocytes play a key role in cerebral development [68]. Emerging research has demonstrated that in the case of CMV infection, increased risk of symptomatic presentation is associated with infection during the first trimester [29]. In addition, infection during the first trimester is also associated with structural brain anomalies including agyria, lissencephaly, ventriculomegaly, diffuse WM abnormalities and cerebellar hypoplasia [29, 68]. Neuroimaging findings of children infected between 18 and 24 weeks of gestation include descriptions of polymicrogyria, less consistently cerebellar hypoplasia, cortical gyral abnormalities and those of the largest WM hyperintensities described in the frontal region [29, 68]. Infection during the third trimester is associated with a higher likelihood of asymptomatic presentation at birth, and MRI findings including, myelin delay /destruction, periventricular cysts, and damaged periventricular WM. Perinatal CMV infection is associated with MRI findings associated with delay in myelin maturation and focal WM injury [68]. The timing of the gestational insult is important in explaining the underlying biological mechanisms leading to the observed neurological findings. Given what is currently known about CMV infection, the study findings, and symptom presentation in all three different age ranges could be explained by the reasoning that the participants in all the studies included a mixture of children infected at different gestational time points. Unlike CMV infection, there is a gap in the literature on the relationship between timing of congenital and perinatal HIV infection or being HEU and, the type and incidence of WM imaging findings. The demonstrated use of MRI in each of CMV and HIV infection (Table 2), suggests that MRI is useful in assessing CNS involvement in congenital and perinatal infections or exposures and can provide a detailed visualisation of cerebral injury.

Furthermore, timing of WM imaging is important because it affects the detection rate of brain imaging abnormalities. Studies, particularly in childhood, which conduct WM imaging at a specific age, or a narrow age range often have a higher detection rate than studies whose neuroimaging time point is across a broader age range [20]. Only 18% of the studies included in this review had their neuroimaging time point at a specific age. Only 30% of the studies had a neuroimaging timepoint with a narrow age range and, more than half of the included studies had a neuroimaging time point with a broader age range. This could provide an additional explanation for why the study findings were heterogenous (Table 1). These scoping review findings suggest the need for more longitudinal cohort research studies assessing the effect of congenital and perinatal infections or exposures with continued neuroimaging time points at a specific ages or narrower age ranges. Such longitudinal cohort studies will be crucial in establishing timing of WM alterations, trend of changes related to appearance, persistence, or disappearance of WM alterations at different age time points.

#### Key imaging features on DTI for the neurological effect of congenital and perinatal infections on WM in paediatric populations

Among the studies where DTI/DWI was used, 60% of the studies reported lower FA values in children with congenital infections including CMV and HIV. Lower FA in the adult literature is generally associated with impaired microstructural integrity. However, in some of the HEU studies FA was higher. Lower FA in HIV infected children and higher FA in HEU was reported in the cerebellar region in both HIV and HEU at the median age group of birth – 3 years. The time period between birth and 3 years is when most of the rapid WM development occurs [64]. Studies assessing WM microstructure development have previously demonstrated that WM changes during birth – 3 years are greater than any substantial changes observed later in life [64, 69]. There are also regional variations in WM microstructure development where early post-mortem studies previously described a posterior-to-anterior and inferior-to-superior pattern of brain maturation for myelination of WM [64, 69, 70]. Diffusion metric values also exhibit considerable regional variations, even when brain maturation is complete. For example, FA values in the CST reach an asymptote of around 0.53–0.54, while maturity in the inferior and superior longitudinal fasciculi occurs around 0.47, and 0.5, respectively [69, 71]. Scoping review findings suggest that reduced FA could be a specific marker for the impairment or damage of WM integrity in perinatal viral infection. However, less is known in early life about the interpretation of the direction of effects of diffusion metrics and what this means in terms of the health of the WM microstructure at these ages. In addition, less is known about the regional variability of diffusion metrics in early life. Further longitudinal studies assessing WM microstructure in HEU populations are needed to further examine long term FA changes and the implications of FA values in relation to the neurological effect of viral infection exposures on WM integrity.

Studies using DTI/DWI to investigate group differences between children with HIV infection and controls, and children who are HEU and controls also reported associations between diffusion metrics and neurocognitive and neurobehavioral assessments. In perinatal viral infection lower FA and higher MD, RD or AD were associated with poor/ lower performance or impairment in neurodevelopmental assessments assessing motor, language and visual performance, and IQ measures [25, 33, 35, 38, 41, 44].

## Conclusion

Early assessment of CNS involvement in congenital and perinatal infections in children is crucial in providing clinicians with the tools necessary to determine the extent and timing of cerebral injury in order for them to develop more comprehensive diagnostic and treatment strategies. The utility of structural MRI and DTI as complementary diagnosis tools in early assessment of congenital CMV infection in paediatric populations has been described. Quantitative studies using structural MRI and DTI have also suggested specific WM tracts that appear to be most vulnerable to HIV infection and HIV exposure. Unlike in CMV studies, this scoping review did not find any studies of other viral infections and exposures comparing the structural MRI and DTI to other neuroimaging and radiological techniques which can be used to assess the developing brain.

### Electronic supplementary material

Below is the link to the electronic supplementary material.

Supplementary Material 1

Supplementary Material 2

## Data Availability

All data generated or analysed during this study are included in this published article [and its supplementary information files].
