# Supplementary material for: Understanding the impact of congenital infections and perinatal viral exposures on the developing brain using white matter magnetic resonance imaging: a scoping review
Source: BMC Med Imaging. 2024 May 23;24:119. doi: 10.1186/s12880-024-01282-9 (PMC11119575; doi:10.1186/s12880-024-01282-9)
Supplement: Supplementary file 1 — Supplementary Material 1 [file 12880_2024_1282_MOESM1_ESM.docx]

**Additional File 1_Scoping review research strategy**

The scoping review methodology was guided by principles laid out in the Arksey and O’ Malley methodological guide for scoping reviews (1). The Arksey and O’ Malley methodological guide was selected as a methodology of reference as it is the gold standard for scoping review methodologies.

*Stage one: Identifying research question*

Conceptualization of the research question and purpose was based on a rapid review of the literature and consultation with research experts with extensive experience in peadiatrics and child health research. The rapid review of literature suggested that neuroimaging studies investigating the impact of viral infections and exposures had been well described in adult populations. However, unlike adult populations, there still remains much to be understood about the impact of viral infections and exposures on the developing child brain. In addition, the rapid review of literature and consultation of experts also suggested that structural MRI was the most commonly used neuroimaging technique in studies assessing the developing brain. Furthermore, the rapid review of literature revealed the broad range of challenges related to neuroimaging in paediatric populations. In addition, the rapid review of literature revealed a need to clearly:

1. Articulate the definitions of key concepts such as the population age range given that the developing brain often has different WM related and myelination stages at different critical age periods.
2. Choose between either focusing on a mixture of human and animal studies or only focusing on human studies given that animal study findings do not often translate to humans.

Refining the research question involved selecting a population and neuroimaging technique.

**Population:** Strictly human children aged between 0 – 18 years old who either have congenital and perinatal viral infections OR were congenitally or perinatally exposed to viral infections.

**Neuroimaging technique:** The neuroimaging techniques of consideration involved WM imaging techniques represented by MRI modalities.

The scoping review research purpose was articulated as follows:

Children in LMICs continue to have a high burden of congenital and perinatal infections and exposures due to the high prevalence of viral infections in LMICs. A greater understanding of the scope of scholarly work related to the impact of congenital and perinatal viral infections or exposures on the developing brain will identify gaps in the research, clarify key concepts, and highlight opportunities to enhance health outcomes for key paediatric populations.

*Stage two: Identifying relevant studies*

A consultation with relevant experts including paediatrics and child health clinicians, and librarians was conducted prior to developing the search strategy. This was in line with Arksey and O’Malley(1), and Levac, Colquhoun and O’Brien(2) ,who suggested that consultation in scoping review methodology was important because incorporation of experts in methods provides content expertise.

The search strategy was developed to be allow for the full time frame(1986 - 2022) and geographic location (worldwide) applicable to the research question, imaging modality(WM imaging techniques), disease type (congenital and perinatal viral infection or exposure) and population of interest(children), in order to stay aligned with the Arksey and O’Malley(2005) framework goal of comprehensiveness. The literature search was primarily conducted on three electronic databases including Web of Science, Scopus and PubMed. Search key terms included:

1. Web of Science: “( white matter imaging OR white matter magnetic resonance imaging OR white matter MR imaging (All Fields) and congenital infection OR perinatal infection (All Fields)”
2. Scopus: “( ALL ( white AND matter AND imaging OR white AND matter AND magnetic AND resonance AND imaging OR white AND matter AND mr AND imaging ) AND ALL ( congenital AND infection OR perinatal AND infection ) )”
3. PubMed: “(white matter imaging OR white matter magnetic resonance imaging OR white matter MR imaging) AND (congenital infection OR perinatal infection)”

In addition, the search was also extended to reference lists of relevant articles which were manually searched.

*Stage three: Study selection*

Following a comprehensive understanding of the scope of the literature an inclusion and exclusion criteria was created which would guide the process of study selection. The articles were screened per the inclusion criteria for full text review: the article must have involved; human participants, children between birth – 18 years old , congenital and perinatal infections or exposures, WM imaging techniques, and written in English. An independent reviewer reviewed final abstracts and full text articles to verify whether the final articles for inclusion met the specified inclusion criteria guided by the research question and purpose. The search strategy and article selection were reported using a PRISMA flow chart. The PRISMA flowchart is accessible as figure 1 in the scoping review article manuscript ready for publication.

*Stage four: Data charting*

The following data were extracted from articles which met the inclusion criteria into Excel: country, study design, congenital and perinatal infection or exposure, neuroimaging time point, WM imaging modality, participant grouping, age and number of participants, study findings and a notes sections with significance/ utility of WM imaging technique cited in the article. The data table columns were later refined to only include only six columns focused on data extracted pertaining to included articles’: references, congenital or perinatal infection or exposure, neuroimaging time point, participant sample size, WM imaging modality and study findings. Data from columns which ended up being excluded from the final collated data table extensively informed the discussion section of the scoping review. The final collated data table is accessible as a table in the scoping review article manuscript ready for publication.

*Stage five : Results collating, summarizing and reporting*

Given the multiple steps involved in stage five, Levac, Colquhoun and O’Brien(2),and Arksey and O’Malley(1), previously suggested: conducting a quantitative descriptive analysis accompanied by a qualitative thematic analysis , reporting results in line with the research question and purpose, and, interpreting study findings in the context of future research, practice and policy. The organization of results for the final scoping review manuscript followed the aforementioned strategy for stage five. This involved segmenting the quantitative and qualitative research findings. This included a quantitative assessment of whether or not there are differences in groups, understanding how the infection impacts the brain, in order to understand the mechanisms of infectious brain pathophysiology. While also including a qualitative assessment of key diagnostic imaging features for the neurological effect of viral infections or exposures in WM. In line with the suggested approach for stage five the scoping review included a general report on the study findings related to impact of congenital and perinatal infections and exposures on the developing brain in paediatric populations at different ages. Study findings were also interpreted in the context of future research and practice with a section in the scoping review focused on discussing the diagnostic value of WM imaging techniques given the scoping review findings. The comprehensive results and discussion sections in line with the suggested approach for stage five are accessible as a results and discussion sections in the scoping review article manuscript ready for publication in Chapter 3 titled: “Utility of white matter neuroimaging in improving understanding of the impact of congenital and perinatal viral infections or exposures on the developing brain.”


*Stage six (optional): Consulting*
Despite the sixth stage being optional, it was very important to include it in this scoping review given the various challenges in neuroimaging research in paediatric populations. During early growth and development, the maturing brain in children changes rapidly and it is chemically and structurally different from the adult brain(3, 4) Interpretation of WM integrity findings in paediatric populations is also a very complex process due to the stage of myelination at critical age points in children. The myelination process is most rapid if the first two years of life where in the first four weeks of life the maturing brain has very high water content in the unmyelinated WM(3). Therefore, unlike WM MRI signal intensities in adults, WM MRI signal intensities in paediatric populations, particularly in the first two years of life are often very similar to those of grey matter. According to Barkovich and Raybaud(4), the aforementioned similarities of MRI signal intensities often result in poor grey-WM matter contrasts in regions of the maturing brain which are incompletely myelinated. These multiple challenges in paediatric neuroimaging necessitated the consultation of research experts in the field of paediatrics and child health, as they could offer invaluable insights on research strategy development, database sources, clinical insights, reference management and paediatric neuroimaging data interpretation perspectives applicable to the scoping review.


References

1. Arksey H, O'Malley L. Scoping studies: towards a methodological framework. International journal of social research methodology. 2005 Feb 1;8(1):19-32.
2. Levac D, Colquhoun H, O'Brien KK. Scoping studies: advancing the methodology. Implementation science. 2010 Dec;5:1-9.
3. Barkovich MJ, Li Y, Desikan RS, Barkovich AJ, Xu D. Challenges in pediatric neuroimaging. Neuroimage. 2019 Jan 15;185:793-801.
4. Vezina G, Barkovich AJ, Raybaud C. Pediatric neuroimaging.2012
