# Supplementary material for: Understanding the impact of congenital infections and perinatal viral exposures on the developing brain using white matter magnetic resonance imaging: a scoping review
Source: BMC Med Imaging. 2024 May 23;24:119. doi: 10.1186/s12880-024-01282-9 (PMC11119575; doi:10.1186/s12880-024-01282-9)
Supplement: Supplementary file 2 — Supplementary Material 2 [file 12880_2024_1282_MOESM2_ESM.docx]

| **Reference** | **Country** | **Congenital infection / Perinatal viral infection or exposure** | **Neuroimaging time-point** | **Participants, (n)= sample size** | **DTI Analysis method** |
| --- | --- | --- | --- | --- | --- |
| 22 | Netherlands | CMVI | 40.4 – 41.7weeks | CMVI: n=21  CMV uninfected: n=61 | Region of interest analysis |
| 29 | South Africa | HIV and HEU | 7 years | HIV+: n=65  HEU: n=19 HUU n=27 | Voxelwise analysis |
| 30 | South Africa | HIV | 9-11 years | HIV+: n=204  Matched controls: n=44 | FSL’s randomise plus a Freesurfer vertex-wise analysis |
| 31 | South Africa | HIV | 5 years | HIV+:n=38 Controls: n=11 | Voxelwise analysis |
| 32 | South Africa | HIV | 9-12 years | HIV+ : n=168  Controls : n=43 | Whole brain analysis |
| 33 | India | HIV and HEU | 8-15 years | HIV+ : n=22  HEU: n=18  HUU: n=8 | Voxelwise analysis |
| 34 | South Africa | HIV on ART | 9-11 years | HIV+(ART < 2years): n= 46  HIV+ (ART > 2years): n= 79 | Region of interest analysis |
| 35 | Netherlands | HIV on cART | 8-18 years | HIV+ : n=28  Controls : n=34 | Region of interest analysis |
| 36 | Thailand | HEU | 5-15 years | HEU: n=30  HUU : n=33 | Analysis of regions where FA was > 0.2 |
| 37 | South Africa | HIV | 5 years | HIV+: n=39  Controls : n=13 | Voxelwise analysis |
| 38 | South Africa | HIV | 6-16 years | HIV+: n=75  Controls : n=30 | 48 white matter tracts analysis |
| 39 | China | HIV | 12-18 years | HIV+ : n=15  Controls : n=26 | Whole brain analysis |
| 40 | South Africa | HEU | 2-4 week old | HEU : n=15  Controls : n=24 | Region of interest analysis |
| 41 | South Africa | HIV | 6 – 15 years | HIV+ : n=50 | Region of interest analysis |
| 42 | South Africa | HIV | 8-12 years | HIV+: n=12 Healthy controls: n=12 | Region of interest analysis |

**Additional file 2: Demographics overview with DTI analysis method**
